# Objective wearable measures correlate with self-reported chronic pain levels in people with spinal cord stimulation systems

**DOI:** 10.1038/s41746-023-00892-x

**Published:** 2023-08-15

**Authors:** Denis G. Patterson, Derron Wilson, Michael A. Fishman, Gregory Moore, Ioannis Skaribas, Robert Heros, Soroush Dehghan, Erika Ross, Anahita Kyani

**Affiliations:** 1 Nevada Advanced Pain Specialists, Reno, NV USA; 2 Goodman Campbell Brain & Spine, Carmel, IN USA; 3 Center for Interventional Pain and Spine, Lancaster, PA USA; 4 Pacific Sports and Spine, Eugene, OR USA; 5 Expert Pain, Houston, TX USA; 6 Spinal Diagnostics, Tualatin, OR USA; 7 Abbott Neuromodulation, Plano, TX USA

**Keywords:** Predictive markers, Outcomes research, Spinal cord diseases, Neuropathic pain, Predictive markers

## Abstract

Spinal Cord Stimulation (SCS) is a well-established therapy for treating chronic pain. However, perceived treatment response to SCS therapy may vary among people with chronic pain due to diverse needs and backgrounds. Patient Reported Outcomes (PROs) from standard survey questions do not provide the full picture of what has happened to a patient since their last visit, and digital PROs require patients to visit an app or otherwise regularly engage with software. This study aims to assess the feasibility of using digital biomarkers collected from wearables during SCS treatment to predict pain and PRO outcomes. Twenty participants with chronic pain were recruited and implanted with SCS. During the six months of the study, activity and physiological metrics were collected and data from 15 participants was used to develop a machine learning pipeline to objectively predict pain levels and categories of PRO measures. The model reached an accuracy of 0.768 ± 0.012 in predicting the pain intensity of mild, moderate, and severe. Feature importance analysis showed that digital biomarkers from the smartwatch such as heart rate, heart rate variability, step count, and stand time can contribute to modeling different aspects of pain. The results of the study suggest that wearable biomarkers can be used to predict therapy outcomes in people with chronic pain, enabling continuous, real-time monitoring of patients during the use of implanted therapies.

## Introduction

Chronic pain is a debilitating condition affecting a widespread population in the United States, estimated at over 50 million American adults^1^. Pain that lasts for more than three to six months is often considered chronic and is influenced by a complex combination of biopsychosocial factors including but not limited to emotional, psychological, physical, and social considerations^2,3^. Spinal cord stimulation (SCS) is an effective treatment option for chronic pain and often leads to pain reduction and improvement in quality of life^4–7^. Response to therapy over time varies from person to person and often requires interactive adjustment of therapy parameters^8^. Thus, appropriate individual selection and long-term monitoring are crucial in optimizing the outcomes of SCS therapy^9,10^.

The condition of a person with chronic pain is usually evaluated through several patient-reported outcome (PRO) measures administrated manually in an in-clinic visit. The current gold standard for evaluating pain is using unidimensional PROs such as the Numerical Rating Scale (NRS) or the Visual Analog Scale (VAS)^11,12^. Efforts to capture multidimensional aspects of chronic pain and treatment effects have historically been done through the addition of other validated PRO questionnaires^13,14^. To capture the more comprehensive and multidimensional effects of pain, people with chronic pain often answer several other questionnaires, such as the Pain Catastrophizing Scale (PCS)^15^, which provides insight into the psychological aspects of pain, Oswestry Disability Index (ODI) for disability and function^16^, and Patient-Reported Outcomes Measurement Information System 29 (PROMIS-29) for global health measure^17^, Patient Health Questionnaire-9 (PHQ-9) for depression^18^, and Patient Global Impression of Change (PGIC)^19^ for the perception of improvement with different therapies. These tools rely on the person’s assessment, which is subject to memory, cognitive, social desirability, and other psychologically influenced response biases. Additionally, there are limitations and the potential for subjective bias on the part of clinical evaluators^20^. An individual’s perception of pain and its effect on daily activities and overall health are hard to capture in a single data point recorded in a clinical visit^20^. However, frequent collection of multiple questionnaires at shorter interval visits is burdensome for people living with chronic pain (and their clinicians), as it requires up to 54 questions across instruments.

To date, there are no established and validated objective measures for assessing pain and its impact on the person’s overall well-being, and objectively quantifying the effect of SCS treatment on reducing chronic pain. Prior research has emphasized the need for improved metrics to better characterize an individual’s response and change in chronic pain levels with neurostimulation therapies^21,22^. Recent advances in the development of wearable technologies enabling objective measurement of movement, physical activity, and function^23–29^, gait and posture^30–34^, neuromuscular and physiological data^10,30–33^, sleep^35,36^, and behavioral assesment^37,38^ have resulted in the emergence of “digital biomarkers” which could be measured outside the physical confines of the clinics avoiding some of the bias introduced in clinic measurements^30–33,39,40^. Many of these biomarkers from wearables have shown a potential to objectively measure different aspects of an individual’s chronic pain and its effect on physical activity, sleep, psychological health, and social participation^27,28,31,35,41^.

Machine learning (ML) has been extensively used in healthcare to provide insights, enhance decision-making, improve patient outcomes, automate workflows, accelerate medical research, and enhance operational efficiency by analyzing large amounts of data^42–44^. Despite recent research highlighting the importance of using machine learning techniques in pain research, previous works have focused on correlating and monitoring symptoms and side effects of pain with digital biomarkers and not necessarily predicting the subject-reported outcomes^45–47^.

Recent advances in wearable technologies and machine learning algorithms provide a promising opportunity for predicting pain and other subjective measurements of pain^48,49^. Previous studies have emphasized machine learning-based classification of pain intensity, but far less attention has been paid to predicting pain and its multi-dimensional effects that are usually captured with multiple patient-reported outcome questionnaires^49^.

Here, we combined objective measures collected from a custom smartwatch application with predictive machine learning algorithms to predict commonly used PROs to measure chronic pain perception. While these objective assessments are not direct measurements of pain, they have the potential to serve as a highly accurate tool to evaluate changes in the quality of life and the level of disability in people with chronic pain and to develop prediction models to measure response to SCS. The goal of this study was to predict pain perception with machine learning models as measured by PROs from objective data measured before and after the SCS implant collected from commercially available smartwatches.

## Results

### Subject participation and compliance

Twenty participants were enrolled as part of this study. Five patients discontinued participation in the study: one participant withdrew consent prior to permanent SCS system implantation, two participants withdrew consent after permanent implantation, one participant’s participation was terminated by the investigator, and one participant was excluded from analysis due to a lack of wearable data. During the study, the median compliance percentage for completing the PROs through the custom application on the iPhone was 88.8% (Interquartile range of 66.6% to 100%). The participants had to input PROs in a separate research phone and were deemed compliant if they completed PROs at least three times during the baseline and once every month after the implant for a duration of 6 months. In addition, a median compliance of 84.7% (Interquartile range of 70.4 to 95.4%) was achieved for using the smartwatch during the study duration. Participants were deemed compliant if they wore the watch for at least 7 days during the baseline and 180 non-consecutive days after the implant. No significant difference was found in compliance between completing PROs on the iPhone and wearing the watch (Wilcoxon rank-sum test). Participants received follow-up phone calls from clinic staff if they missed providing data for more than 3 consecutive days or completing PROs on the custom application. The average age of participants was 52.25 (±9.7) years at baseline. On average, all participants suffered from 12 years of chronic pain. Back pain was the primary pain diagnosis of the majority of the participants (85%) in the presented cohort. Table 1 summarizes the baseline characteristics of the study participants.

**Table 1:** Baseline characteristics of study participants.

Baseline Characteristics	*N* (%) or mean (± SD)
**Gender**	
*Male*	15 (75%)
*Female*	5 (25%)
**Age**	
*Years*	52.25 (±9.77)
**Pain Duration**	
*Years*	12.3 (±11.7)
**Etiology**	
*Radiculopathy*	5 (25%)
*Non-surgical Back Pain*	4 (20%)
*Failed Back Surgery Syndrome*	8 (40%)
*Complex Regional Pain Syndrome Type I*	3 (15 %)

*SD* standard deviation.

### SCS therapy improves pain, function, and quality of life in people with chronic pain

**Fig. 1:**
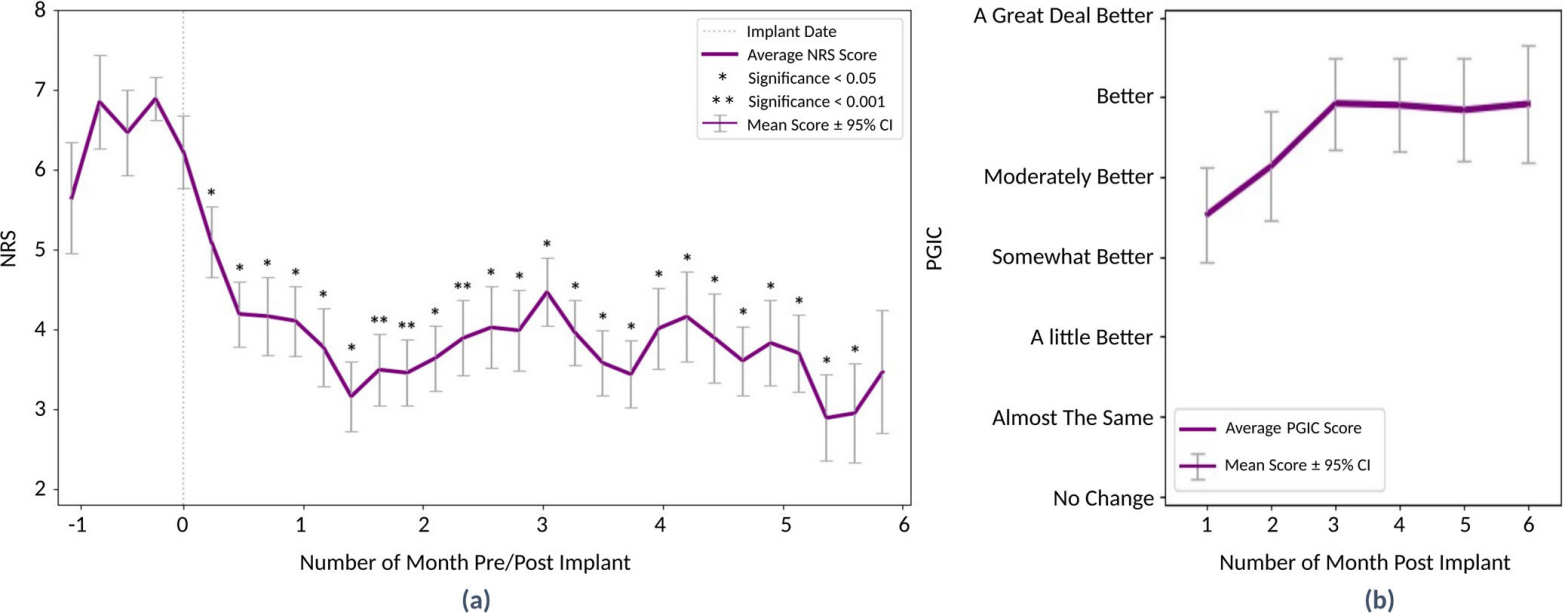
Improvement in PROs collected from the custom wearable application. **a** The weekly average of NRS scores across all participants during the study; 0 indicates the permanent implant date; the baseline period values (−1 to 0 month) are the weekly average of NRS pulled from the watch application; statistical testing indicates a comparison to pre-implant pain scores per subject and is calculated using the Wilcoxon signed-rank test on median values; *p ≤ 0.05, **p ≤ 0.001. **b** Monthly average of PGIC scores for all participants during the study; NRS numerical rating scale, PGIC patient global impression of change, CI confidence intervals.

During the course of the treatment, participants showed improvements in NRS and all other PROs collected through the wearable application and in-clinic visits (for comparison of the baseline visit to the last pre-op in-clinic visit). A summary of all PROs at different time points and a comparison of in-clinic and application data is presented in Table 2. The average and standard deviation of in-clinic NRS values dropped from 7.2 (±0.88) at baseline to 3.14 (±1.83), and 3.34 (±2.12) at the 3- and 6-month visits, respectively. Moreover, the average daily NRS collected from the custom watch application was reduced across all participants (Fig. 1a). Similar improvements were seen for all PROs included in this study (Table 2). Figure 1b shows improvement seen in PGIC across all participants based on monthly reported values on the custom iPhone application. The raw scores for the PROs collected using the wearable application and in-clinic visits at baseline, 3-months, and 6-months show significant improvement in PROMIS-29’s sleep disturbance, social roles, pain interference, and fatigue compared to baseline (Table 2). The raw scores for the PCS and ODI showed significant improvements compared to baseline as well. The improved trend for the values collected through the digital health custom wearable application is like the improvement trend in the values collected during clinic visits. The sample size for each comparison is listed for each measurement. All participants must complete PROs in the clinic as part of the required case report forms for the study.

**Table 2:** Scores for PROs collected in the study.

	Score					
PRO	Baseline In Clinic	Baseline In App	3-month In Clinic	3-month In App	6-month In Clinic	6-month In App
NRS	7.2 ± 0.8 (*n* = 15)	6.6 ± 1.5 (*n* = 15)	3.1 ± 1.8 * (*n* = 14)	3.5 ± 2.1 ** (*n* = 15)	3.3 ± 2.1 * (*n* = 15)	3.4 ± 1.9 * (*n* = 10)
PGIC	N/A	N/A	5.7 ± 1.4^a^ (*n* = 14)	5.1 ± 1.9^a^ (*n* = 8)	5.0 ± 1.7^a^ (*n* = 15)	4.8 ± 2.0^a^ (*n* = 10)
PCS	25.2 ± 11.5 (*n* = 15)	26.9 ± 10.6 (*n* = 14)	13.5 ± 11.9 * (*n* = 14)	10.1 ± 8.8 * (*n* = 10)	18.5 ± 15.9 (*n* = 15)	14.0 ± 11.7 * (*n* = 10)
PHQ-9	N/A	10.5 ± 5.6 (*n* = 14)	N/A	4.0 ± 3.6 (*n* = 7)	N/A	4.8 ± 3.8 (*n* = 9)
ODI	51.6 ± 15.8 (*n* = 15)	56.0 ± 15.9 (*n* = 13)	32.0 ± 16.9 * (*n* = 14)	29.8 ± 20.6 * (*n* = 9)	34.7 ± 18.0 * (*n* = 14)	33.5 ± 20.1 * (*n* = 9)
PR-29 - Physical Function	33.5 ± 4.5 (*n* = 15)	32.5 ± 3.9 (*n* = 14)	40.4 ± 6.6 * (*n* = 13)	33.1 ± 6.7 (*n* = 8)	41.9 ± 8.5 * (*n* = 15)	32.4 ± 4.9 (*n* = 8)
PR-29 - Social Roles	38.1 ± 6.3 (*n* = 15)	37.4 ± 6.5 (*n* = 15)	47.0 ± 7.8 * (*n* = 13)	47.9 ± 9.9 * (*n* = 10)	46.6 ± 10.2 (*n* = 15)	44.8 ± 10.9 (*n* = 10)
PR-29 - Depression	51.7 ± 7.0 (*n* = 15)	56.9 ± 8.5 (*n* = 15)	51.3 ± 7.1 (*n* = 13)	50.3 ± 7.2 (*n* = 10)	50.7 ± 8.9 (*n* = 15)	52.7 ± 10.2 (*n* = 10)
PR-29 - Anxiety	50.5 ± 17.8 (*n* = 15)	55.5 ± 16.1 (*n* = 15)	45.1 ± 18.5 (*n* = 13)	38.7 ± 17.2 (*n* = 10)	41.7 ± 18.9 (*n* = 15)	39.6 ± 18.9 (*n* = 10)
PR-29 - Fatigue	60.3 ± 6.6 (*n* = 15)	61.5 ± 7.1 (*n* = 15)	53.1 ± 8.11 * (*n* = 13)	50.6 ± 5.6 * (*n* = 10)	54.4 ± 10.9 * (*n* = 15)	52.8 ± 9.3 (*n* = 10)
PR-29 - Sleep Disturbance	58.7 ± 7.2 (*n* = 15)	55.7 ± 3.7 (*n* = 15)	50.8 ± 8.3 * (*n* = 13)	49.8 ± 3.1 * (*n* = 10)	49.2 ± 10.4 * (*n* = 15)	50.0 ± 3.8 * (*n* = 10)
PR-29 - Pain Interference	67.9 ± 6.1 (*n* = 15)	68.2 ± 5.7 (*n* = 15)	59.3 ± 8.3 * (*n* = 13)	57.1 ± 8.3 * (*n* = 10)	58.2 ± 8.4 * (*n* = 15)	60.0 ± 10.7 (*n* = 10)

Scores are shown as mean ± standard deviation (Number of participants); The significance is calculated using the two-sided Wilcoxon signed-rank test on median values for in-app data throughout that month and every single value for in-clinic data; The asterisk shows the statistically significant differences (*p ≤ 0.05; **p ≤ 0.001).

*NRS* numerical rating scale, *PR-29* patient-reported outcomes measurement information system 29, *PCS* pain catastrophizing scale, *PHQ-9* patient health questionnaire-9, *ODI* Oswestry disability index, *PGIC* patient global impression of change, *SD* standard deviation.

^a^The categorical values of PGIC are shown as numbers from 1 to 7 (low improvement to high improvement).

### Objective data can be used to passively monitor and predict daily pain level

The physiological and behavioral features collected passively throughout the study were used to construct a machine learning model to predict daily categorical pain levels in the participants. A variety of different machine learning models were attempted for predicting three categorical levels of pain intensity, with the random forest model yielding the best predictive performance. Specifically, the random forest model showed high accuracy in predicting three intensity levels of mild, moderate, and severe pain, corresponding to NRS levels of <4, ≥4 & ≤6, and >6, respectively (F1 Score = 0.768 ± 0.012 and Accuracy = 0.768 ± 0.012, Sensitivity = 0.737 ± 0.016, and Specificity = 0.869 ± 0.007) (Table 3, Supplementary Fig. 1). This model was driven using objective features as an input and could theoretically be used to passively monitor daily pain intensity categories in people with chronic pain.

**Table 3:** Evaluation metrics for machine learning modeling of PROs using objective features.

PRO	Accuracy (mean ± SD)	F1 Score (mean ± SD)	Sensitivity (mean ± SD)	Specificity (mean ± SD)	Precision (mean ± SD)	AUC – ROC (mean ± SD)
NRS	0.768 ± 0.012	0.768 ± 0.012	0.737 ± 0.016	0.869 ± 0.007	0.775 ± 0.021	0.889 ± 0.032
PGIC	0.827 ± 0.063	0.821 ± 0.058	0.657 ± 0.094	0.904 ± 0.059	0.709 ± 0.169	0.873 ± 0.081
PCS	0.837 ± 0.025	0.796 ± 0.048	0.600 ± 0.284	0.935 ± 0.057	0.744 ± 0.158	0.893 ± 0.076
PHQ-9	0.852 ± 0.030	0.835 ± 0.037	0.620 ± 0.198	0.913 ± 0.045	0.703 ± 0.121	0.864 ± 0.061
ODI	0.697 ± 0.042	0.657 ± 0.045	0.667 ± 0.053	0.852 ± 0.029	0.701 ± 0.075	0.862 ± 0.064
PR-29 - Physical Function	0.884 ± 0.001	0.830 ± 0.001	0.978 ± 0.029	0.533 ± 0.339	0.942 ± 0.041	0.784 ± 0.173
PR-29 - Social Roles	0.817 ± 0.035	0.812 ± 0.038	0.664 ± 0.110	0.896 ± 0.057	0.796 ± 0.082	0.883 ± 0.014
PR-29 - Depression	0.887 ± 0.054	0.884 ± 0.053	0.687 ± 0.115	0.893 ± 0.047	0.646 ± 0.0966	0.888 ± 0.027
PR-29 - Anxiety	0.843 ± 0.045	0.839 ± 0.046	0.766 ± 0.110	0.888 ± 0.037	0.755 ± 0.067	0.915 ± 0.032
PR-29 - Fatigue	0.864 ± 0.067	0.850 ± 0.076	0.678 ± 0.078	0.920 ± 0.058	0.746 ± 0.142	0.889 ± 0.041
PR-29 - Sleep Disturbance	0.948 ± 0.001	0.923 ± 0.001	0.350 ± 0.390	0.970 ± 0.032	0.437 ± 0.390	0.877 ± 0.102
PR-29 - Pain Interference	0.846 ± 0.061	0.845 ± 0.061	0.833 ± 0.095	0.816 ± 0.078	0.844 ± 0. 0.058	0.917 ± 0.032

*AUC-ROC* area under the receiver operating characteristic (ROC) curve, *NRS* numerical rating scale, *PR-29* patient-reported outcomes measurement information system 29, *PCS* pain catastrophizing scale, *PHQ-9* patient health questionnaire-9, *ODI* Oswestry disability index, *PGIC* patient global impression of change, *SD* standard deviation.

### Objective data can be used to passively monitor and predict other aspects of pain

To holistically predict the well-being of people with chronic pain, we used the objective measures collected from the custom watch app to predict categories of common PROs for chronic pain assessments. We developed 11 machine learning models to predict score categories from PROs collected throughout the study (PGIC, PCS total score, 7 domains of PROMIS-29, ODI total score, PHQ-9 total score) using objective features. The results of PRO modeling and evaluation metrics for each of the PROs are shown in Table 3. All models had high accuracy and F1 score indicating the application of digital biomarkers in predicting categories of these subjective measures for chronic pain. In addition to pain intensity, these models could be used to predict different dimensions of pain including emotional aspects using depression, anxiety, and fatigue (PROMIS-29, PHQ-9) models, physical function aspects using physical activity (PROMIS-29) model and sleep aspects using sleep disturbance (PROMIS-29) model. The PCS model could help with predicting pain catastrophizing in people with chronic pain while the PGIC model could help with predicting individual response to therapy and be used as a tool to find the right candidates for spinal cord stimulation. Most models achieved high sensitivity and specificity, but the datasets for PROMIS-29 domains of physical function and sleep disturbance were highly imbalanced. This was resolved by use of the synthetic minority oversampling technique (SMOTE). However, a remaining limitation was the limited number of data points in minority classes and a lack of sleep data from the smartwatch as an input for predicting sleep disturbance. This limitation likely explains the low specificity for physical function and low sensitivity for the sleep model (Table 3).

### Important biomarkers for pain

**Fig. 2:**
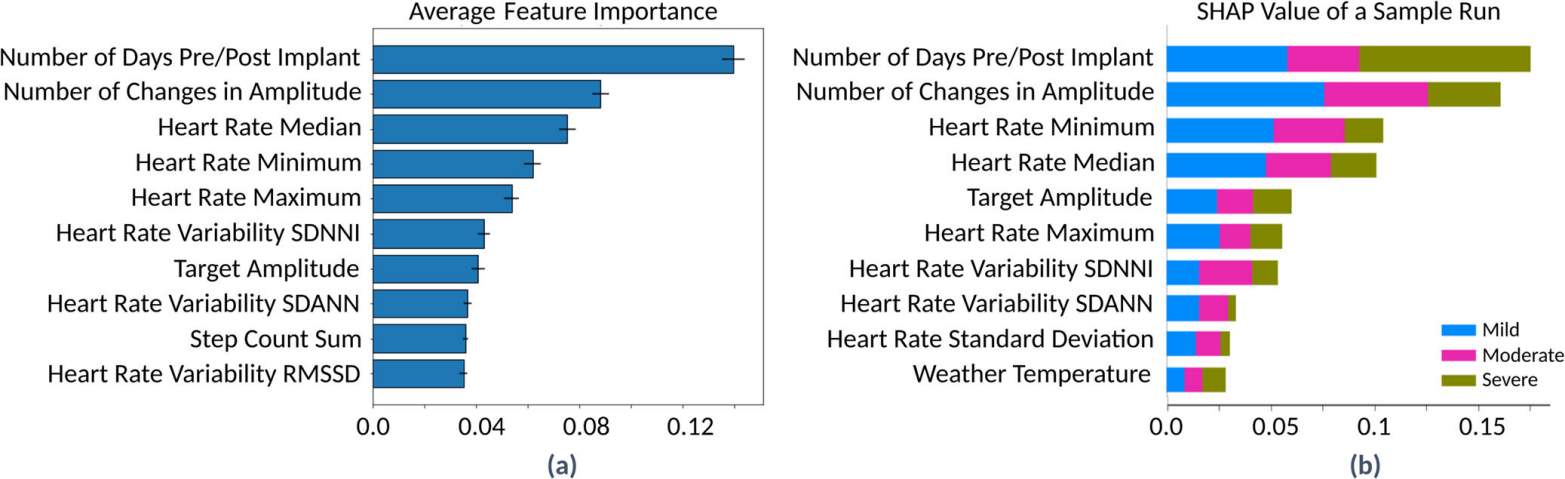
Important features from ML modeling for pain. **a** The average of feature importance for modeling pain levels using objective data across 10 runtimes. The standard deviation is shown with a line in the bar plot. **b** A sample of SHAP (SHapley Additive exPlanations) values for one run time. RMSSD: The root mean square of successive inter-beat intervals of heartbeats differences. SDANN: The standard deviation of the average inter-beat intervals without artifacts for each 5-minute interval over a 24-hour recording of heart rate variability. SDNNI: The average of the standard deviations of all inter-beat intervals without artifacts for each 5-minute interval over a 24-hour recording of heart rate variability.

The feature importance for modeling three levels of pain intensity and SHAP (SHapley Additive exPlanations) values are plotted in Fig. 2. The figure includes the feature importance for the model built with objective features (a and b). Several wearable biomarkers such as heart rate, step count, and stand time were shown to be important predictors of pain and various aspects of it. The feature importance analysis of the model using objective biomarkers depicts those physiological biomarkers from the Apple^®^ Watch that played an important role in modeling three categorical levels of pain (e.g., heart rate, heart rate variability, step count, and stand time). The number of days pre/post implant was also found to be a prominent feature for predicting pain, likely due to the time it takes for SCS therapy to be adjusted to optimal settings post-implant. The device programming features that were pulled from the patient’s SCS controller app were also among the important features. Participants who reported more variation of pain score had a higher engagement with the patient controller app (i.e., they visited the patient controller application more often to adjust therapy settings).

**Fig. 3:**
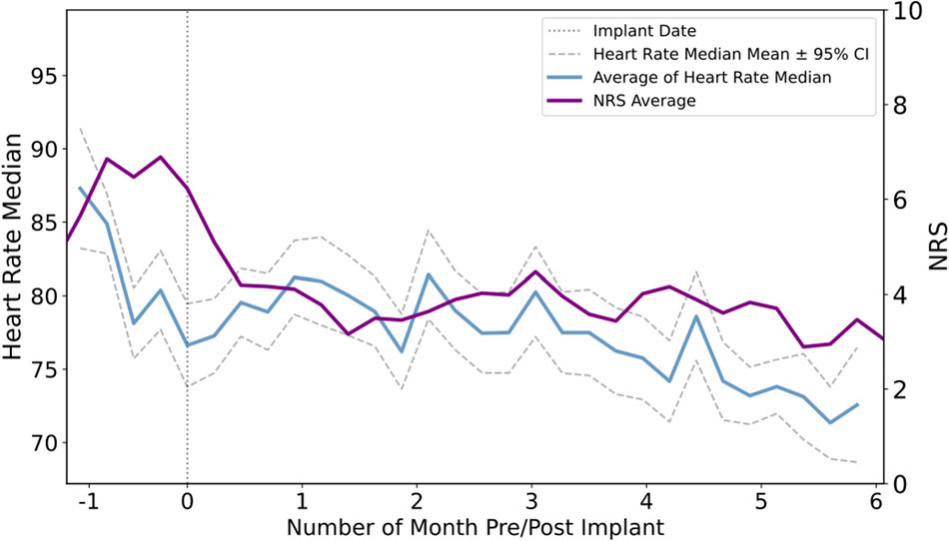
The average of heart rate median values correlates with the NRS score. The weekly average of heart rate median values across all participants during the study along with the weekly average of NRS scores; NRS numerical rating scale, CI confidence intervals.

Furthermore, there was a correlation between the median heart rate (as an important feature) and pain intensity demonstrating that on days with lower pain levels, participants experienced lower heart rate values during the day (Fig. 3)^50^.

The feature analysis for the other predictive models for other aspects of pain showed the same pattern as the predictive model for pain levels, and features such as heart rate, heart rate variability, step count, and stand time were identified as top predictors. The number of months post-implant also appeared as one of the top features in most of the PRO models, suggesting a gradual wash-in of the therapy effect over time. Moreover, these results from pain and PRO modeling indicated that heart rate variability can contribute to the understanding of pain. Further investigations showed that participants with lower pain intensity have a higher heart rate variability which is seen in people with chronic pain^41,51^. Finally, the total number of programming changes, the target stimulation amplitude (from programming data), and the local weather temperature and precipitation were among the top predictors in most of PRO models. This suggests that the programming setting and weather may influence the daily pain level reported by patients^52–54^.

## Discussion

SCS treatment is an effective therapy that can provide much-needed pain relief, improve physical activity and social participation, and ultimately enhance the quality of life for people dealing with chronic pain. Here we report, the results of this research which further support the efficacy of SCS by showing an improvement in average pain relief post-implant across participants accompanied by average improvement in the quality-of-life metrics including physical activity, social roles anxiety, fatigue, sleep disturbance, and pain interference. Objective measures to monitor each individual’s progress over the period of treatment could help with increasing therapeutic efficacy and better adoption of new technologies. Our approach in this paper is to highlight the feasibility of using objective data from a wearable device to not only create a model for predicting categorical pain intensity but also to predict other outcome quality-of-life outcome measures typically measured in clinics. These results suggest that a machine learning model can use passive data to predict and categorize participants based on NRS, PCS, PROMIS-29 domains, ODI, PHQ-9, and PGIC. This is an important and distinct improvement over just categorizing participants using unidimensional scales.

The smartwatch compliance that passively collected participant’s physiological signals highlights the importance of wearables in new technology adoption. The collection of PROs through the custom application and use of a smartwatch provides frequent data required for validating these predictive models. The objective data from wearables can be used to develop predictive algorithms for long-term passive monitoring of symptoms and reducing the burden of completing PROs in the future. It is noteworthy to mention that patients in this study are selected based on their comfort level with technology and willingness to engage in digital health activities. The study’s device compliance rates may overestimate real-world use because patients are actively monitored, and clinical staff call them if they miss completing PROs or providing data for more than three days.

There are distinguishing differences in the features extracted from the Apple^®^ Watch during the baseline period compared to post-SCS treatment across all participants. These differences indicate physical and physiological changes in people with chronic pain which are measured using a wearable watch. For example, in the physical aspect of pain, the average total stand time increases after implant across all participants which suggests higher activity and social engagement in people with chronic pain treated with SCS. Additionally, the average daily stand time follows the NRS improvement in participants, suggesting that better pain relief leads to a higher average standing time throughout the day. The predictive models accurately predict three levels of daily pain and various aspects of pain captured by commonly used and validated PROs using objective data as inputs. The feature importance analysis reveals activity metrics, heart rate, and heart rate variability as important predictors of pain. The number of days pre/post implant is another crucial feature, indicating that participants take time to experience a lower pain level, and the change in pain intensity is not immediate. This suggests that participants experience varying levels of pain relief depending on the timing of their SCS implants until their pain levels reach a more stable state. Monitoring of patient data using passive means may help in the future by informing optimization of settings with a closed-loop SCS system and choosing the appropriate window to change the SCS configuration based on the pain level.

Chronic pain often affects other dimensions of an individual’s life such as sleep, physical function, psychological health, and quality of life. The study outcomes demonstrate improvement of different aspects of pain in people with chronic pain after spinal cord stimulation therapy and the potential use of wearables to capture these measures objectively since different pain domains such as physical function, social behavior, and sleep can be quantified through wearable sensing^55–57^. The predictive models of PROs developed in this study could be used to monitor an individual’s progress through the SCS continuum and decrease the burden of completing PROs in the app or the clinic. The predictive model for PGIC developed with high accuracy can be used for patient selection and to provide therapy to people with chronic pain for whom SCS is more effective.

The main strength of this work is developing predictive models to predict pain and other aspects of it using objective data. We develop a large set of biomarkers and build accurate and robust models that could be used to characterize pain and well-being in people with chronic pain. One limitation of the current study is the small sample size for developing machine learning models which can affect the generalizability of our predictions given the variability across different patients. To mitigate this, we randomize the training and testing data 10 times and report the average model performance. The short-term application of this work is a population model that can be personalized to each patient using some of their initial data. But in the longer term, with more data and a more diverse dataset, we may be able to generate population models that operate without personalization.

Another limitation of the study is the lack of reliable sleep data as an important predicting factor for pain, due to the inadequate time resolution and the fact that only binary values are provided for sleep data. Additionally, the amount of physical activity information is limited for this version of the watch compared to future generations and clinically validated sensors which could affect our ability to predict pain levels and categories of PRO measures. Moreover, the imbalanced datasets for PROMIS-29 physical function, and sleep disturbance with a few data points in the minority class limit us in building robust models with high specificity and sensitivity in predicting these two domains of PROMIS-29.

There are multiple confounding factors such as post-surgery recovery time, inactivity due to surgery, effects of medication, and other factors that can affect the interpretation of wearable features selected (e.g., heart rate, step count) for this study. Including daily averages and variations of the features as input to the model could decrease such effects. In addition, the pain modeling is performed using data up to 6 months post-implant to have a more robust prediction. The validity of all the features of the model needs to be further studied to better understand the nature of the relationship between the predictor and a patient’s pain level and to rule out potential confounding factors. This study is designed to demonstrate feasibility in a small patient population but future studies including a larger sample size of people with chronic pain are required to overcome these limitations. In addition, improvements in future generations of wearable devices can provide access to additional sensors and data; resulting in helping with the robustness of predictive models aimed at solving complex modeling of individual’s pain.

In summary, with increasing the availability of both consumer and research-grade wearable devices and accessing sophisticated machine learning techniques, the opportunity of developing novel methods to passively monitor the daily changes in people with chronic pain and predict their pain states becomes more achievable. The ability to identify patients passively with waning therapy, or other painful clinical events (such as a fall), would help bring them back in for evaluation and thereby drive improvement in their long-term outcomes. Wearables can objectively measure many features which are influenced by participants’ chronic pain such as activity, sleep, psychological health, and social participation. Adding objective measurements could improve the accuracy of classification models and enable us to move toward a more personalized therapy with a limited burden on both people with a chronic pain and clinicians.

## Methods

### Study design and baseline characteristics

Data were extracted as a sub-study of the prospective, multicenter, international REALITY (Long-Term Real-World Outcomes Study on Patients Implanted with a Neurostimulator) study (NCT03876054; March 15, 2019). Prior to initiating the study, Western Institutional Review Board approval was received for the study sites and all participants were provided with written informed consent. The study inclusion criteria for REALITY were designed with few restrictions on the pain indication as allowed by the regulatory bodies in each geographical region and according to standard clinical practice to replicate the range of complex participants that would be seen in everyday medical practice. The sub-study was designed to compare changes in pain and physical function, and behavioral markers before and 6 months after SCS implantation. The sub-study participants were asked to wear a smartwatch and answer multiple PROs frequently on a custom-designed application in addition to the regular in-clinic visits. Study visits occurred at enrollment, baseline, three- and six-month post-implantation.

### Data collection

Demographics were collected at baseline and included the duration of pain, work status, exercise level, and the number of previous surgeries. PRO measures to assess pain intensity (NRS), physical function and disability (ODI), emotional distress and depression (PCS), and global health (PROMIS-29 and PGIC) were collected at baseline and each follow-up study visit. All sub-study participants were provided with an Apple ® Watch (Series 3) at enrollment. Participants were prompted to enter NRS scores collected on the investigational custom watch application daily from baseline until six months after the implant. The watch application was given access to HealthKit data and passively collected several HealthKit metrics for activity, behavior, and cardiac measures such as heart rate, heart rate variability, step count, stand time, and distance walking/running. The subject needed to access the watch application at least once a day. Once participants selected their current pain level from 0 (no pain) to 10 (the worst pain imaginable) in the custom watch application, the app then sent the NRS data to a secured cloud storage. The watch application is an iOS-based application that pulls Healthkit data from the Apple Watch. As soon as a user rates the pain intensity on the UI, the app is activated, and physical activity and heart rate data are collected in the background. The UI screen shows the elapsed time from the start of the data collection to the subject. The REALITY iPhone custom application is a companion to the watch application and installation of the REALITY wearable application on the iPhone will automatically install the watch application on the paired Apple® Watch. The iPhone application is an iOS-based application to collect behavioral data from subjects. PROs such as PROMIS-29, ODI, PCS, and PHQ-9 were collected on a regular basis through the phone application (3 times before implant and once every month after the implant for a period of 6 months). PGIC was collected monthly for 6 months after the implant.

### Statistical analysis

The normality of PROs data was assessed using the Shapiro-Wilk test. The two-sided Wilcoxon signed-rank test was used to measure the significance of the change in PROs pre- versus post-implant on median values. The two-sided Wilcoxon rank-sum test for the independent non-normal sample was used to measure the significance of compliance with completing PROs on the phone and the watch application. *P*-values less than 0.05 are considered as the significance level.

### Data preprocessing and featurization

Apple^®^ HealthKit provided features for step counts, stand time, walking/running distance, sleep, heart rate and heart rate variability, and the number of flights climbed. Post-processing of the data showed that there are a high number of missing values for sleep and flights climbed acquired through the HealthKit app. We removed these measures for further analysis in this manuscript. We used a threshold that was calculated based on the number of data points recorded each day to discard days with inadequate data points, or sparse data. Specifically, a threshold was determined by using 5% of the median value derived from the daily number of data points within the same pain level. We removed data from days that had fewer data points than the threshold. To balance feature weights and handle missing data for low-resolution features on the Apple® Watch, we used a daily window for data points with the same pain level in our analyses. Statistical features such as maximum, minimum, sum, mean, standard deviation, 25^th^, 75^th^, and 90^th^ percentiles were extracted from the daily windowed data. Furthermore, since there were many missing data points in the heart rate variability (HRV) of the Apple^®^ HealthKit data and the inter-beat interval was not accessible, heart rate was used to estimate this time interval in order to calculate HRV in three different methods, 1) the root mean square of successive inter-beat intervals of heartbeats differences (RMSSD), 2) the standard deviation of the average inter-beat intervals without artifacts (NN intervals) for every 5 min over a 24 h-period of HRV recording (SDANN), and 3) mean of the standard deviations of all the NN intervals for every 5 min over a 24 h-period of HRV recording (SDNNI)^58^.

Different data streams were used and aggregated for comprehensive analyses and to provide a deeper understanding of digital biomarkers contributing to participant’s therapy outcomes. The SCS device programming information was also pulled from the patient’s controller application (Abbott, Plano, TX). However, programming data from the patient controller application on the phone had missing values due to retention policies. Therefore, we imputed programming data using the minimum value for each programming feature. Additionally, we used publicly available datasets for weather information (https://www.ncei.noaa.gov/) based on the location of participants, moon phase (https://www.timeanddate.com/), and the stock market data of three popular stocks, NASDAQ Composite, DIJA, and S&P 500 (https://finance.yahoo.com/). The stock market data was also imputed when the market was closed, and the price on the last day was used to fill the missing prices. The features with high correlation (>0.90) were removed from the dataset and the remaining features were used for developing machine learning models.

### Predictive models using machine learning

The machine learning models were developed on Apple^®^ HealthKit data, programming data, and other features discussed in the data collection and featurization section. Daily NRS values and weekly scores for each PRO were used as the main output variables for the predictive machine learning models.

Several ML techniques such as Logistic Regression^59^, Support Vector Machine^60^, K-Nearest Neighbors^61^, Random Forest^62^, and Catboost^63^ were implemented on the dataset for predicting daily pain values as well as other PROs and their performance was compared on the testing data, and random forest, a tree-based model, provided the best performance and interpretability of features (Supplementary Table 1). Additionally, SHAP (SHapley Additive exPlanations)^64,65^ technique was used to calculate the contribution of features. Of the total data available, 80% of the data was used for training and 20% for the testing phase.

**Fig. 4:**
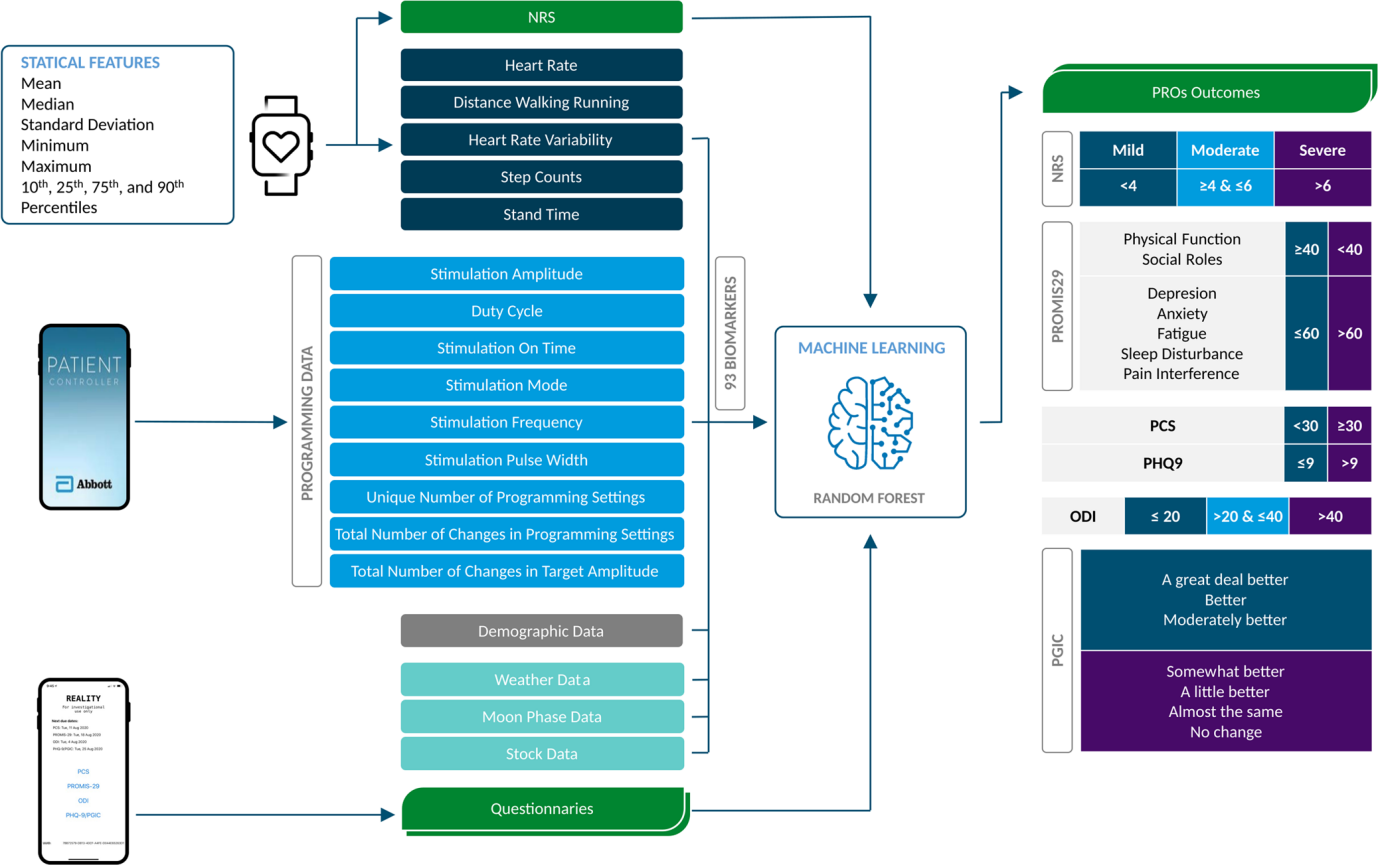
Feature engineering and predictive modeling pipeline. The patient-reported outcomes (PROs) of numerical rating scale (NRS), patient-reported outcomes measurement information system 29 (PROMIS-29), pain catastrophizing scale (PCS), patient health questionnaire-9 (PHQ-9), Oswestry disability index (ODI), patient global impression of change (PGIC) are outputs of machine learning models.

The random forest model was developed using digital biomarkers collected from the Apple^®^ Watch, programming data from the patient controller, and other features such as weather data based on the participant’s primary residence zip code. To increase the robustness of predictions among the training sets, the random forest model was trained 10 times using randomly selected 80% of the input data available. The reported outcomes were then averaged across all 10 different runs. Figure 4 shows the machine learning pipeline for predicting pain and other PRO measurements.

A balanced number of training sets for each class of output variable were considered and NRS and other PROs were grouped into different classes. We categorized NRS values into three groups: mild (NRS < 4), moderate (NRS ≥ 4 and NRS ≤ 6), and severe (NRS > 6) pain; PROMIS-29 into two groups, physical function, and social roles were grouped as responders (T-score ≥ 40) and non-responders (T-score < 40), depression anxiety, fatigue, sleep disturbance, and pain interference into two groups of responders (T-score ≤ 60), and non-responders (T-score > 60);^14,66^ PCS into two groups of catastrophizing (total score ≥ 30), and non-catastrophizing (total score < 30); PHQ-9 into two groups of responders (total score ≤ 9) and non-responders (total score > 9)^18^, ODI into three classes of high responders (≤ 20), low responders (> 20 & ≤ 40), and non-responders (> 40);^67^ PGIC, into two groups of responders (Moderately better, Better, and A great deal better) and non-responders (No change, Almost the same, A little better, and Somewhat better). The synthetic minority oversampling technique (SMOTE)^68^ was used to address imbalanced datasets for PROMIS-29 physical function and sleep disturbance for building their predictive models.

### Reporting summary

Further information on research design is available in the Nature Research Reporting Summary linked to this article.

### Supplementary information

Supplementary information

Reporting Summary

## Data Availability

Data used in this study may be made available to qualified individuals for collaboration if a written request is made to and granted in writing by Abbott at Abbott’s sole discretion. The requester should include their name, title, contact information, and the institution they work for as well as the specifics regarding the use and necessity of the requested dataset to the corresponding author. Abbott retains full discretion over its data and is under no obligation, legal or otherwise, to release or provide it to third parties regardless of the request being made.

## Code availability

The codes for data processing, modeling, and visualization were developed using open-source Python libraries including pandas version 1.5.0, scikit-learn version 1.0.2, scipy version 1.9.1, numpy version 1.21.5, and matplotlib version 3.5.3. The code may be made available to qualified individuals for collaboration if a written request is made to and granted in writing by Abbott at Abbott’s sole discretion. The requestor should include their name, title, contact information, and the institution they work for as well as the specifics regarding the use and necessity of requested codes to the corresponding author. Abbott retains full discretion over its code and is under no obligation, legal or otherwise, to release or provide it to third parties regardless of the request being made.
