# Supplementary material for: Objective wearable measures correlate with self-reported chronic pain levels in people with spinal cord stimulation systems
Source: NPJ Digit Med. 2023 Aug 15;6:146. doi: 10.1038/s41746-023-00892-x (PMC10427619; doi:10.1038/s41746-023-00892-x)
Supplement: Supplementary file 1 — Supplementary information [file 41746_2023_892_MOESM1_ESM.pdf]

Supplementary Information

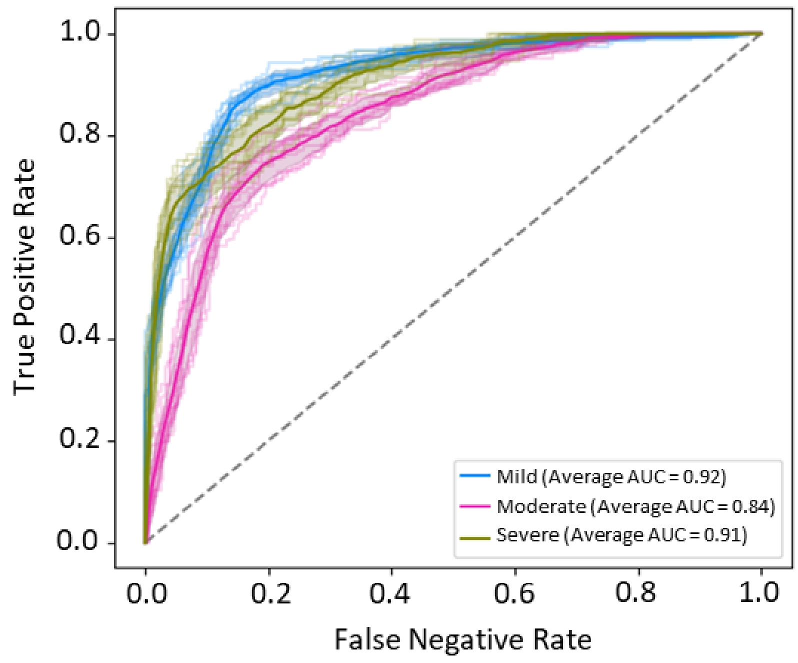

Supplementary Fig. 1: The receiver operating characteristic (ROC) curve for the pain model using objective data; The ROC curve of each run time is plotted. The average of 10 runtimes for each class is shown in a solid bold line with the standard deviation around it (Thin lines); AUC area under the ROC curve.

Supplementary Table 1 Results of pain modeling using other machine learning techniques

**Model**	**Accuracy**	**F1 Score**	**Sensitivity**	**Specificity**	**Precision**	**AUC - ROC**
**RF**	0.768 (0.012)	0.768 (0.012)	0.737 (0.0167)	0.869 (0.007)	0.775 (0.021)	0.889 (0.032)
CB	0.733 (0.015)	0.729 (0.014)	0.696 (0.0169)	0.852 (0.008)	0.743 (0.016)	0.870 (0.037)
SVM	0.684 (0.017)	0.681 (0.016)	0.655 (0.0190)	0.830 (0.008)	0.664 (0.021)	0.832 (0.045)
KNN	0.654 (0.021)	0.646 (0.023)	0.608 (0.0232)	0.810 (0.011)	0.647 (0.027)	0.807 (0.043)
LR	0.602 (0.011)	0.592 (0.014)	0.543 (0.0198)	0.779 (0.007)	0.587 (0.017)	0.753 (0.037)

The results of average models are shown as mean (standard deviation) across 10 different runtimes. The tested ML models are Random Forest (RF), Catboost (CB), Support Vector Machine (SVM), K-Nearest Neighbors (KNN), and Logistic Regression (LR); AUC-ROC area under the receiver operating characteristic (ROC) curve.
