# Supplementary material for: Objective wearable measures correlate with self-reported chronic pain levels in people with spinal cord stimulation systems
Source: NPJ Digit Med. 2023 Aug 15;6:146. doi: 10.1038/s41746-023-00892-x (PMC10427619; doi:10.1038/s41746-023-00892-x)
Supplement: Supplementary file 2 — Reporting Summary [file 41746_2023_892_MOESM2_ESM.pdf]

nature portfolio

Corresponding author(s): Dr. Denis G. Patterson

Last updated by author(s): 2023/05/26

# Reporting Summary

Nature Portfolio wishes to improve the reproducibility of the work that we publish. This form provides structure for consistency and transparency in reporting. For further information on Nature Portfolio policies, see our Editorial Policies and the Editorial Policy Checklist.

## Statistics

For all statistical analyses, confirm that the following items are present in the figure legend, table legend, main text, or Methods section.

| n/a | Confirmed | |
|---|---|---|
| ☐ | ☒ | The exact sample size (*n*) for each experimental group/condition, given as a discrete number and unit of measurement |
| ☐ | ☒ | A statement on whether measurements were taken from distinct samples or whether the same sample was measured repeatedly |
| ☐ | ☒ | The statistical test(s) used AND whether they are one- or two-sided<br>*Only common tests should be described solely by name; describe more complex techniques in the Methods section.* |
| ☒ | ☐ | A description of all covariates tested |
| ☐ | ☒ | A description of any assumptions or corrections, such as tests of normality and adjustment for multiple comparisons |
| ☐ | ☒ | A full description of the statistical parameters including central tendency (e.g. means) or other basic estimates (e.g. regression coefficient) AND variation (e.g. standard deviation) or associated estimates of uncertainty (e.g. confidence intervals) |
| ☐ | ☒ | For null hypothesis testing, the test statistic (e.g. *F*, *t*, *r*) with confidence intervals, effect sizes, degrees of freedom and *P* value noted<br>*Give P values as exact values whenever suitable.* |
| ☒ | ☐ | For Bayesian analysis, information on the choice of priors and Markov chain Monte Carlo settings |
| ☒ | ☐ | For hierarchical and complex designs, identification of the appropriate level for tests and full reporting of outcomes |
| ☒ | ☐ | Estimates of effect sizes (e.g. Cohen's *d*, Pearson's *r*), indicating how they were calculated |

*Our web collection on statistics for biologists contains articles on many of the points above.*

## Software and code

Policy information about availability of computer code

| | |
|---|---|
| Data collection | We developed a custom application on Apple Watch and iPhone to collect physiological and behavioral data from the participants throughout the study. The watch application is an iOS-based application that pulls Healthkit data from the Apple Watch. As soon as a user rates the pain intensity on the UI, the app gets active and physical activity and heart rate data are collected in the background. The UI screen shows the elapsed time from the start of the data collection to the subject. The REALITY iPhone custom application is a companion to the watch application and installation of the REALITY wearable application on the iPhone will automatically install the watch application on the paired Apple Watch. |
| Data analysis | All codes were developed using open source Python libraries such as Pandas=1.5.0, Scikit-learn=1.0.2, Scipy=1.9,1, Numpy=1.21.5 Matplotlib=3.5.3. |

For manuscripts utilizing custom algorithms or software that are central to the research but not yet described in published literature, software must be made available to editors and reviewers. We strongly encourage code deposition in a community repository (e.g. GitHub). See the Nature Portfolio guidelines for submitting code & software for further information.

## Data

Policy information about availability of data

All manuscripts must include a data availability statement. This statement should provide the following information, where applicable:

- Accession codes, unique identifiers, or web links for publicly available datasets
- A description of any restrictions on data availability
- For clinical datasets or third party data, please ensure that the statement adheres to our policy

The data used for building models would be available upon request.

## Research involving human participants, their data, or biological material

Policy information about studies with human participants or human data. See also policy information about sex, gender (identity/presentation), and sexual orientation and race, ethnicity and racism.

| | |
|---|---|
| Reporting on sex and gender | 15 male and 5 female participants were enrolled in the study. The study was not powered to make any specific conclusions based on gender or sex. |
| Reporting on race, ethnicity, or other socially relevant groupings | Race was an optional question on the informed consent form. The study was not powered to make any specific conclusions based on race and ethnicity. |
| Population characteristics | The average age of participants was 52.25 (±9.7) years at baseline. On average, all participants suffered from 12 years of chronic pain and back pain was the primary pain diagnosis of the majority of the participants (85%) in the presented cohort. The baseline demographic and medical information of the enrolled subjects have been provided in the manuscript (Table 1). |
| Recruitment | Participants were recruited through clinical sites. We had 6 clinical sites in this study to enroll participants. A subject is considered enrolled in the study when they have provided written informed consent of the study and they have been determined to meet all Inclusion/Exclusion requirements. |
| Ethics oversight | Western Institutional Review Board (WIRB) |

Note that full information on the approval of the study protocol must also be provided in the manuscript.

# Field-specific reporting

Please select the one below that is the best fit for your research. If you are not sure, read the appropriate sections before making your selection.

☒ Life sciences ☐ Behavioural & social sciences ☐ Ecological, evolutionary & environmental sciences

For a reference copy of the document with all sections, see nature.com/documents/nr-reporting-summary-flat.pdf

# Life sciences study design

All studies must disclose on these points even when the disclosure is negative.

| | |
|---|---|
| Sample size | 20 participants |
| Data exclusions | Exclusion criteria has been used and explained in details in the protocol. In total, data from five subjects were excluded; one participant withdrew consent prior to permanent SCS system implantation; two participants withdrew consent after permanent implantation; one participant's participation was terminated by the investigator; one participant was excluded from analysis due to a lack of wearable data. |
| Replication | This was a prospective, multicenter, non-randomized, single arm, open-label feasibility study and replication was not relevant to the study. |
| Randomization | This was a prospective, multicenter, non-randomized, single arm, open-label feasibility study. |
| Blinding | This was a prospective, multicenter, non-randomized, single arm, open-label feasibility study and blinding was not relevant to the study. |

# Reporting for specific materials, systems and methods

We require information from authors about some types of materials, experimental systems and methods used in many studies. Here, indicate whether each material, system or method listed is relevant to your study. If you are not sure if a list item applies to your research, read the appropriate section before selecting a response.

## Materials & experimental systems

| n/a | Involved in the study | |
|---|---|---|
| ☒ | ☐ | Antibodies |
| ☒ | ☐ | Eukaryotic cell lines |
| ☒ | ☐ | Palaeontology and archaeology |
| ☒ | ☐ | Animals and other organisms |
| ☐ | ☒ | Clinical data |
| ☒ | ☐ | Dual use research of concern |
| ☒ | ☐ | Plants |

## Methods

| n/a | Involved in the study | |
|---|---|---|
| ☒ | ☐ | ChIP-seq |
| ☒ | ☐ | Flow cytometry |
| ☒ | ☐ | MRI-based neuroimaging |

# Clinical data

Policy information about clinical studies

All manuscripts should comply with the ICMJE guidelines for publication of clinical research and a completed CONSORT checklist must be included with all submissions.

| | |
|---|---|
| Clinical trial registration | NCT03876054 |
| Study protocol | The study protocol and informed consent form have been submitted to the journal. |
| Data collection | Data collection has been detailed in both the manuscript and the protocol. Enrollment duration was about one year, and subjects were followed up for six months after permanent implant. The total duration of the sub-study was about two years, including enrollment, data collection from all subjects, and sub-study close out. |
| Outcomes | This study was an exploratory, prospective, multicenter, non-randomized, single arm and open-label data collection study. The data was used to develop machine learning algorithms to predict pain intensity and patient's reported outcomes in people with chronic pain. There was no statistically powered endpoint. |
